# An Improved Natural Transformation Protocol for the Cyanobacterium *Synechocystis* sp. PCC 6803

**DOI:** 10.3389/fpls.2020.00372

**Published:** 2020-04-15

**Authors:** Matthew A. Pope, Josh A. Hodge, Peter J. Nixon

**Affiliations:** Sir Ernst Chain Building-Wolfson Laboratories, Department of Life Sciences, Imperial College London, London, United Kingdom

**Keywords:** ploidy, natural transformation, flow cytometry, cyanobacteria, phosphate depletion

## Abstract

The naturally transformable cyanobacterium *Synechocystis* sp. PCC 6803 is a widely used chassis strain for the photosynthetic production of chemicals. However, *Synechocystis* possesses multiple genome copies per cell which means that segregating mutations across all genome copies can be time-consuming. Here we use flow cytometry in combination with DNA staining to investigate the effect of phosphate deprivation on the genome copy number of the glucose-tolerant GT-P sub-strain of *Synechocystis* 6803. Like the PCC 6803 wild type strain, the ploidy of GT-P cells grown in BG-11 medium is growth phase dependent with an average genome copy number of 6.05 ± 0.27 in early growth (OD_740_ = 0.1) decreasing to 2.49 ± 0.11 in late stationary phase (OD_740_ = 7). We show that a 10-fold reduction in the initial phosphate concentration of the BG-11 growth medium reduces the average genome copy number of GT-P cells from 4.51 ± 0.20 to 2.94 ± 0.13 and increases the proportion of monoploid cells from 0 to 6% after 7 days of growth. In addition, we also show that the DnaA protein, which unusually for bacteria is not required for DNA replication in *Synechocystis*, plays a role in restoring polyploidy upon subsequent phosphate supplementation. Based on these observations, we have developed an alternative natural transformation protocol involving phosphate depletion that decreases the time required to obtain fully segregated mutants.

## Introduction

Cyanobacteria are photoautotrophic prokaryotes capable of converting light into chemical energy via oxygenic photosynthesis. This characteristic has spurred interest in using cyanobacteria as solar-powered microbial cell factories to produce a variety of carbon-based chemicals (Angermayr et al., 2015; Sengupta et al., 2018).

*Synechocystis* sp. PCC 6803 (hereafter *Synechocystis*) is widely used as a model cyanobacterium for fundamental and applied research because it is naturally transformable (Grigorieva and Shestakov, 1982; Kufryk et al., 2002), can grow heterotrophically on glucose (Anderson and McIntosh, 1991), and has a publicly available genome sequence (Ikeuchi, 1996; Morris et al., 2014; Tichý et al., 2016). Exogenous DNA molecules introduced into *Synechocystis* via natural transformation may replicate independently on a suitable plasmid (Mermet-Bouvier et al., 1993) or be incorporated into the genome via double homologous recombination (Grigorieva and Shestakov, 1982; Shestakov et al., 1985). As such *Synechocystis* is widely used in the field of biotechnology with engineered strains producing a variety of industrially relevant chemical commodities (Xue and He, 2018; Katayama et al., 2018; Gale et al., 2019).

The genome of *Synechocystis* consists of a circular chromosome and seven plasmids (Kaneko et al., 2003). Like many other prokaryotes (Pecoraro et al., 2011; Soppa, 2011), *Synechocystis* is polyploid meaning it possess multiple genome copies per cell (Griese et al., 2011; Tichý et al., 2016; Zerulla et al., 2016). Initial reports on wild-type *Synechocystis* sp. PCC 6803 (Labarre et al., 1989) suggested 12 chromosome copies per cell but the presence of up to 142 copies during exponential growth was reported for a glucose-tolerant strain (Griese et al., 2011). These discrepancies might be a consequence of growth conditions, differences in sub-strains or the techniques used to measure genome copy number.

The molecular details underpinning the regulation of DNA replication and ploidy in cyanobacteria remain unclear although ploidy has been positively correlated with cell size and internal protein concentration (Zheng and O’Shea, 2017) and is influenced by phosphate availability (Zerulla et al., 2016). Unusually, DNA replication in *Synechocystis* is asynchronous and does not occur at a defined origin (Ohbayashi et al., 2015), and the DnaA protein, which is an essential DNA replication initiation factor in most bacteria (Messer, 2002; Katayama et al., 2010), is not crucial for replication (Richter et al., 1998; Ohbayashi et al., 2015).

One disadvantage of polyploidy is that it impedes the construction of mutants. Mutations introduced into the genome must present themselves in all genome copies before a mutant phenotype is revealed or maintained stably. Normally, antibiotic-resistance cassettes are used to select for positive transformants, which will initially possess both wild-type and mutated genomes. Re-streaking single colonies on antibiotic-containing medium is then used to segregate mutations across all genome copies. This segregation process typically takes several weeks and can be a bottleneck in the construction of mutant strains. Non-lethal genetic disruptions may be fully segregated after two rounds of selection, however, if essential loci are disrupted, fully segregated mutants may never be obtained. The time required to generate fully segregated mutants varies in each laboratory and is dependent the cyanobacterial background-strain and the type of mutation (Lea-Smith et al., 2016).

Here we have tested the feasibility of improving the segregation process in *Synechocystis* by identifying conditions in which the number of genome copies in the recipient cell is reduced. This was achieved by systematically examining the degree of ploidy as a function of phosphate availability in the medium and the growth phase. Cells were stained with the DNA specific fluorochrome, SYBR Green I, and flow cytometry was used to determine the DNA content of individual cells within a large population by comparing the fluorescence intensity from each cell to that of monoploid *E. coli* cells containing a known amount of DNA (4.6 Mbp), a method used previously by Majerník et al. (2005) for *Methanothermobacter thermautotrophicus* and Tichý et al. (2016) for *Synechocystis*.

Our approach confirms that the degree of ploidy is dependent on phosphate availability and furthermore identifies a physiological role for DnaA in determining the extent of ploidy and the rate with which polyploidy is restored following the addition of phosphate after phosphate deprivation. Based on our observations, we have developed a revised natural transformation protocol involving cells pre-grown in low-phosphate medium that reduces the time required to obtain fully segregated mutants.

## Materials and Methods

### Bacterial Strains and Culture Conditions

The following strains were used: the glucose-tolerant GT-P sub-strain of *Synechocystis* sp. PCC 6803 (Tichý et al., 2016), *E. coli* K-12 MG1655 (Blattner et al., 1997), and *E. coli* 10-β (New England BioLabs). *E. coli* was grown in M9 minimal medium supplemented with glucose (Supplementary Tables 1–5).

*Synechocystis* GT-P was grown in either liquid BG-11 medium (Rippka et al., 1979) or on BG-11 agar plates (Stanier et al., 1979) at 31°C and pH 8.2 {buffered with 1 mM N-[Tris(hydroxymethyl)methyl]-2-aminoethanesulfonic acid (TES)} under continuous white fluorescent illumination of 40 μmol photons m^–2^s^–1^. Three different types of BG-11 medium, differing in the amount of added phosphate, were used: conventional BG-11 (referred to as “high-phosphate”) containing 175 μM K_2_HPO_4_, “low phosphate” containing 17.5 μM K_2_HPO_4_ or “no-phosphate” with no added phosphate. The composition of high-phosphate, low-phosphate and no-phosphate BG-11 growth media can be found in Supplementary Tables 6–17. Antibiotics were present in the following concentrations: kanamycin (10–50 μg ml^–1^), chloramphenicol (20 μg ml^–1^), and ampicillin (100 μg ml^–1^) (Supplementary Table 18).

### Generation of *Synechocystis* GT-P Δ*dnaA* and Δ*psbD1/C* Mutants

To assemble the Δ*dnaA* gene-deletion construct, an InFusion Cloning Kit (Takara Biosciences) was used to generate a linear DNA molecule consisting of a chloramphenicol-resistance cassette (PCR amplified from pBAD33 vector) flanked by 500 bp of DNA upstream and downstream of the *dnaA_sll0848* open reading frame of *Synechocystis* GT-P. The primers used to amplify each DNA part were DnaA_H1_F (5′-TTTAGCCAATCGTCG AGGAC-3′) and DnaA_H1_R (5′-ACCCCTGGCGATCGGCGA TTATGAG-3′) for the upstream region; primers CamR_DnaA_F (5′-CATAATCGCCGATCGCCAGGGGT**TGATCGGCACGTA AGAGGTTCCAAC**-3′) and CamR_DnaA_R (5′-CACCAATAG AGTTTTCCTACTAAGT**GGCGTTTAAGGGCACCAATAACT GC**-3′) for the chloramphenicol-resistance cassette and primers DnaA_H2_F (5′-ACTTAGTAGGAAAACTCTATTGGTG-3′) and DnaA_H2_R (5′-ATTGTCCAAAGTCTCTGGGC-3′) for the downstream region, with sequences in bold depicting regions homologous to the chloramphenicol-resistance cassette.

The final linear DNA molecule was ligated into the pGEM-T vector (Promega A137A) to generate plasmid pDnaAKO_Cam^R^ which was used to transform *Synechocystis* GT-P via natural transformation.

The Δ*psbD1/C* gene deletion construct was kindly provided by Dr. Jianfeng Yu of Imperial College London.

### Genotyping

Deletion of the *dnaA_sll0848* open reading frame was confirmed by single colony PCR (Supplementary Figure 1). A single colony was picked and resuspended in 50 μl of distilled deionized water (hereafter _dd_H_2_O) in a 200 μl PCR tube. The cell suspension was placed in 80°C water for 30 s then dipped into liquid nitrogen for 5 s. This process was repeated 10 times. The PCR tube was then centrifuged to pellet cell debris. 1 μl of the supernatant was used as template for the PCR genotyping reaction. MyTaq 2X MasterMix (BioLine) was used with primers DnaA_seq_F (5′-TCTCATAATCGCCGATCGCCA-3′) and DnaA_seq_R (5′-GAGATAACCACTCGCACTTGC-3′). A 10 μl reaction was prepared consisting of 1 μl supernatant, 0.2 μl forward primer (final concentration 10 μM), 0.2 μl reverse primer (final concentration 10 μM), 5 μl MyTaq 2X MasterMix, and 3.6 μl _dd_H_2_O. Reaction conditions were as follows: 2 min at 98°C then 30 cycles of 15 s at 98°C, 30 s at 60°C, and 30 s at 72°C followed by 5 min at 72°C.

Deletion of the *psbD1/C* cluster was confirmed by single colony PCR as described above using primers *psbD1/C*_Forward (5′-TAGACCCCTGGCGATCGGCGATTAT-3′) and *psbD1/C*_Reverse (5′-TTGCCAAAGTATTCTCCTGAT TTAAATGATATTGAGCA-3′).

### Preparation of *E. coli* Reference Cells

A single colony was picked from a LB agar plate and used to inoculate 5 ml of M9 minimal medium. Cells were grown by shaking (100 rpm) at 21°C for 7 days. 100 μl of cell culture was used to inoculate another 5 ml of M9 minimal medium and grown at 21°C for a further 7 days. 20 μg ml^–1^ of chloramphenicol was then added to the cell culture to inhibit protein biosynthesis (Dinos et al., 2016) and cells were incubated for a further 60 min. 100 μl of the cell culture were then harvested by centrifugation and fixed in ice-cold 70% ethanol/H_2_O and stored at −20°C.

### Flow Cytometric Analysis of DNA Stained Cells

50 μl of cells were collected by centrifugation from a liquid culture (OD_740_ ≤ 1 for *Synechocystis* GT-P and OD_600_ ≤ 1 for *E. coli*), resuspended in ice-cold 70% (v/v) ethanol and stored in the dark at −20°C for up to 3 weeks. Subsequently, ethanol-fixed *Synechocystis* or *E. coli* cells were pelleted by centrifugation. The pellet was washed twice in TE Buffer (10 mM Tris, 1 mM EDTA, pH 8.0) and resuspended to a final concentration of 1 × 10^7^ cells ml^–1^ (OD_740_ of 0.1). 1 μl of RNAse A (10 mg ml^–1^) was added to the cells and incubated at 37°C for 1 h. SYBR Green I DNA stain (ThermoFisher Scientific S7585) was added at a 1:10,000 dilution in TE buffer and cells were covered in foil and incubated at room temperature for 30 min.

All analyses were conducted on a BD Biosciences LSR II cytometer. Three biological replicates of each strain were analyzed. 96-well flat bottom plates were used to load samples onto the cytometer with 200 μl of stained cells in each well. Control samples containing filter-sterilized TE buffer and unstained *Synechocystis* GT-P and *E. coli* cells were used to confirm that the population of incidents were desired cells. SYBR Green I stained cells were excited with 488 nm light and the emission spectra were captured in the 530/30 nm channel. Genome copies were allocated to DNA frequency peaks as described by Mori et al. (1996). All analysis of flow cytometric data was conducted on FlowJo^TM^ software as described in Supplementary Figures 2, 3.

### Natural Transformation of *Synechocystis* GT-P

#### Phosphate-Deprivation Protocol

10 ml of low-phosphate liquid BG-11 medium without glucose was inoculated with GT-P or Δ*dnaA Synechocystis* cells, obtained from low-phosphate BG-11 liquid cultures, to a starting OD_740_ of 0.03. Cells were grown under 40 μmol photons m^–2^ s^–1^ of fluorescent white light, shaking at 100 rpm at 31°C in air for 7 days. An appropriate volume of cells was collected by centrifugation in a 2 ml eppendorf tube, washed twice in no-phosphate BG-11 and resuspended in 1 ml of no-phosphate BG-11 to give a final OD_740_ of 1. Cells were collected again by centrifugation and 1 μg of Δ*psbD/C* plasmid DNA (Supplementary Figure 4) was added to the tube. No-phosphate BG-11 was added to a final volume of 50 μl. Cells were resuspended and left in low light (10–20 μmol photons m^–2^ s^–1^) in 31°C for 6 h, with agitation every hour by flicking the tube.

After 6 h the 50 μl cell suspension was spread onto a 0.45 μm nitrocellulose filter membrane (Whatman) and placed onto a no-phosphate BG-11 agar plate containing 5 mM D-glucose. The plate was incubated at 31°C under 40 μmol photons m^–2^ s^–1^ of fluorescent white light for 24 h. After 24 h the filter membrane was transferred to a no-phosphate BG-11 agar plate containing 10 μg ml^–1^ of kanamycin and 5 mM D-glucose and left to incubate similarly for 7 days. The filter membrane was then transferred to a low-phosphate BG-11 agar plate containing 10 μg ml^–1^ of kanamycin and 5 mM D-glucose and left to incubate until single colonies appeared. Colonies were re-streaked once on low-phosphate BG-11 agar plates (25 μg ml^–1^ of kanamycin) with 5 mM glucose. We then screened single colonies by testing for photoautotrophic growth on high-phosphate BG-11 agar plates (25 μg ml^–1^ of kanamycin) without glucose. Cells were incubated at 31°C under 40 μmol photons m^–2^ s^–1^ of fluorescent white light for 7 days prior to PCR genotyping.

### Conventional Protocol

The conventional protocol is described as above except each step was conducted with high-phosphate BG-11 and *Synechocystis* cells used for transformation were obtained from an exponentially growing culture (OD_740_ of 0.1–0.3).

### Statistical Analyses

To test the effect of phosphate availability on genome copy number in GT-P and Δ*dnaA Synechocystis* cells, a linear mixed modeling approach was used (Breslow and Clayton, 1993; Pinheiro and Chao, 2006). The response variable was fit using the natural log of the fluorescence intensity (FI in arbitrary units) of the DNA stained cells. The fixed factors were *phosphate condition* (three levels; high-, low-, and no-phosphate), *sampling time* (five levels: Day 2, 7, 21, 26, and 28) and cell type (two levels: Δ*dnaA* and GT-P). A three-way interaction model was fitted in order to estimate the mean fluorescence of the cell type in a phosphate condition at a particular sampling time of interest. The data set consisted of multiple observations of the same cell cultures (3 for Δ*dnaA* and 3 for GT-P), therefore *replicate* was fitted as a random intercept. Model assumptions were checked, and outliers screened. To examine the differences between conditions, least squares means were compared using pairwise *t*-tests with a Bonferroni correction to adjust for multiple testing. A *t*-value greater than 16.25 or lower than −16.25 was interpreted as a significant difference. All statistical analyses were conducted in the R environment (R Development Core Team, 2011).

## Results

### Dependence of Genome Copy Number on Phosphate Availability

Previous work using quantitative PCR (qPCR) to determine genome copy number has described a correlation between environmental phosphate concentration and genome copy number in the original motile wild-type *Synechocystis* PCC 6803 strain: after 7 days of growth in BG-11 medium prepared with 0.13 mM phosphate, the average genome copy number remained unchanged (10 ± 1 to 10 ± 2) while growth in the absence of phosphate decreased the average genome copy number from 10 ± 1 to 4 ± 1 (Zerulla et al., 2016).

Given the potential problems of using qPCR to determine copy number, we opted to use flow cytometry to quantify the genome copy number of a glucose-tolerant sub-strain of *Synechocystis* (GT-P) (Tichý et al., 2016) with the added advantage that it would allow us to examine to what extent the concentration of environmental phosphate affected genome copy number on a single cell basis. *Synechocystis* cells were incubated with the fluorochrome SYBR Green I (Life Technologies), which binds stoichiometrically to dsDNA molecules (Zipper et al., 2004; Clarindo and Carvalho, 2011). Stained cells were excited at 497 nm and the intensity of fluorescence at 520 nm recorded. *E. coli* MG1655 cells grown in minimal medium with a doubling time of ≥16 h and then treated with chloramphenicol were used as the reference as monoploid cells enriched under these conditions contain a genome size of 4.6 Mbp (Skarstad et al., 1983) (Supplementary Figures 2, 3). Assuming 1:1 ratios of plasmids (total size 0.38 Mbp) to chromosome (3.57 Mbp), the *Synechocystis* GT-P monoploid genome is approximately 3.95 Mbp, or 86% the size of the *E. coli* genome, in excellent agreement with the observed ratio of average fluorescence intensities of 0.86:1 assigned to monoploid *Synechocystis* GT-P and *E. coli* cells, respectively (Supplementary Table 22).

**FIGURE 1:**
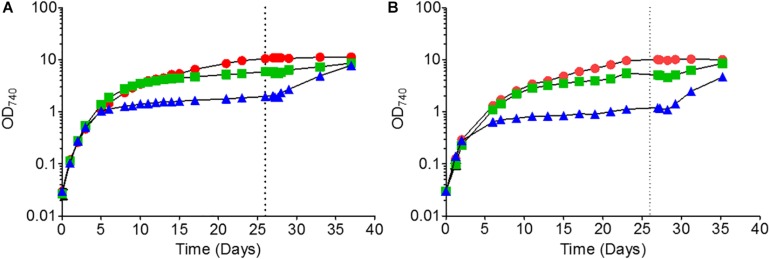
Growth of **(A)** *Synechocystis* GT-P and **(B)** *Synechocystis* Δ*dnaA* in high, low and no-phosphate BG-11. The average OD_740_ of three biological replicates is shown. Error bars are smaller than the size of the symbols. The vertical dashed line at 624 h indicates the time point where a further 175 μM K_2_HPO_4_ was added to the cultures. Cells were grown under 40 μmol photons m^–2^ s^–1^ of white light at 100 rpm and 31°C.

For each time course experiment, *Synechocystis* cells were grown in liquid BG-11 medium containing 0, 17.5 or 175 μM of added K_2_HPO_4_ (termed no-phosphate, low-phosphate and high-phosphate, respectively). For *Synechocystis* GT-P, all three cultures exhibited equivalent growth rates over the first 4 days with a doubling time of approximately 24 h (Figure 1A). However, the final OD achieved by each culture was different, with cultures grown in the absence of added phosphate giving a final OD_740_ of 1.8 compared to OD_740_ of 6 and OD_740_ of 9 for low and high-phosphate BG-11, respectively (Figure 1A). The ability of *Synechocystis* to grow in the absence of added phosphate has been observed previously (Zerulla et al., 2016) and is probably due to their ability to use stored phosphate in the form of polyphosphate bodies as well as DNA (Morohoshi et al., 2002; Van De Meene et al., 2006). The addition of 175 μM K_2_HPO_4_ after 26 days led to renewed growth for both the low and no-phosphate cultures, but not for high-phosphate, suggesting that phosphate was limiting for these cultures (Figure 1A).

**FIGURE 2:**
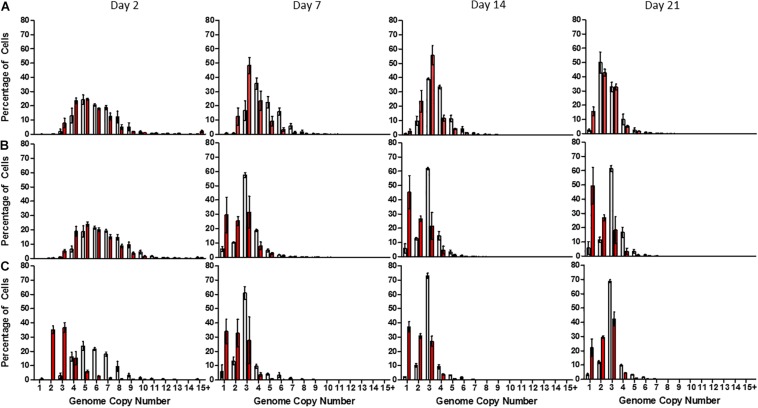
Distribution of genome copy number in populations of *Synechocystis* GT-P (gray) and *Synechocystis* Δ*dnaA* (red) after 2, 7, 14, and 21 days growth in **(A)** high phosphate **(B)** low phosphate, and **(C)** no-phosphate BG-11. The average genome distribution of three biological replicates are shown with error bars. The *x*-axis portrays the genome copy number per cell and the *y*-axis the percentage of cells within a sample of 50,000 cells. Cells were grown under 40 μmol photons m^–2^ s^–1^ of white light, at 100 rpm and 31°C.

Linear mixed modeling (Supplementary Table 19) and least square means analyses (Supplementary Tables 20–22) were used to interpret the flow cytometry data and generate estimated average genome copy numbers within a 95% confidence interval. After 2 days of growth, cells exhibited an estimated average genome copy number of 6.05 ± 0.27 (median: 5), 6.51 ± 0.29 (median: 5), and 5.76 ± 0.25 (median: 5) when grown in high, low and no-phosphate BG-11, respectively, with a maximum value of 16 and minimum value of 2 (Figures 2A–C). After 7 days of growth, cells grown in high-phosphate BG-11 possessed on average 4.51 ± 0.2 (median: 4) genome copies with no cells exhibiting monoploidy (Figure 2A) whereas cells grown in low (Figure 2B) and no-phosphate BG-11 (Figure 2C) exhibited significantly fewer genome copies with average values of 2.94 ± 0.13 (median: 3) and 2.91 ± 0.13 (median: 3), respectively, with 6% of the population monoploid.

**FIGURE 3:**
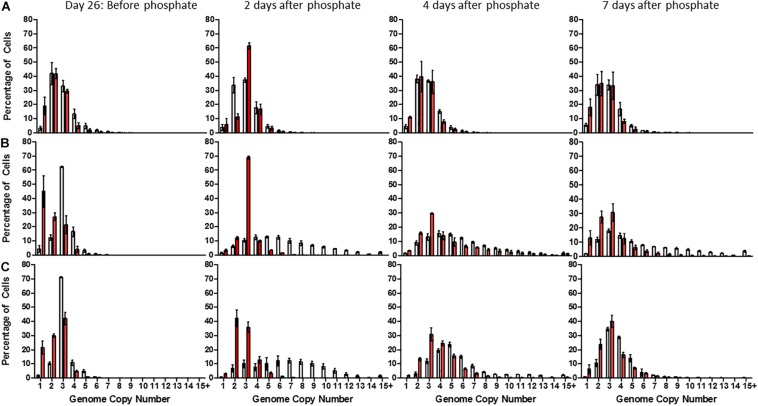
Distribution of genome copy number in populations of *Synechocystis* GT-P (gray) and *Synechocystis* Δ*dnaA* (red) after 26 days growth in **(A)** high phosphate **(B)** low phosphate, and **(C)** no-phosphate BG-11 and 2, 4, and 7 days after phosphate supplementation. The average genome distribution of three biological replicates are shown with error bars. The *x*-axis portrays the genome copy number per cell and the *y*-axis the percentage of cells within a sample of 50,000 cells. Cells were grown under 40 μmol photons m^–2^ s^–1^ of white light, at 100 rpm and 31°C.

48 h after phosphate supplementation, high-phosphate cultures maintained an average genome copy number of 2.81 ± 0.12 (median: 3) (Figure 3A). However, low and no-phosphate cultures exhibited a significant increase in average genomes from 2.84 ± 0.13 (median: 3) to 5.7 ± 0.25 (median: 5) and 2.93 ± 0.13 (median: 3) to 6.13 ± 0.27 (median: 7), respectively, with each population displaying an almost even distribution of 2–12 copies (Figures 3B,C). These data suggest that DNA replication and cell division are rapidly induced upon addition of phosphate to the medium.

### DnaA Plays a Role in Controlling Ploidy

Previous work has shown that the DnaA protein is not needed for DNA replication in *Synechocystis* and that a *dnaA* null mutant grows as well as WT in BG-11 medium (Richter et al., 1998; Katayama et al., 2010; Ohbayashi et al., 2015). However, the effects on growth and ploidy under phosphate limiting conditions have not been examined.

We found that an independently constructed *dnaA* null mutant made in the GT-P background (Supplementary Figure 1) did indeed grow as well as GT-P when grown in high and low-phosphate BG-11 but showed a slight difference when grown under no-phosphate, exhibiting stationary phase at an OD_740_ of 0.8 compared to an OD_740_ of 1.2 for GT-P (Figures 1A,B). The response of the Δ*dnaA* cells to the addition of 175 μM of K_2_HPO_4_ at 26 days was not significantly different to that of GT-P.

The average genome copy number of Δ*dnaA* cells was 5.34 ± 0.23 (median: 5) and 5.59 ± 0.25 (median: 5) per cell when measured 2 days after inoculation into high and low-phosphate BG-11, compared to 6.05 ± 0.27 (median: 5) (high-phosphate) and 6.51 ± 0.29 (median: 5) (low-phosphate) for GT-P (Figures 2A,B). However, Δ*dnaA* cells grown in no-phosphate BG-11 exhibited an average genome copy of 2.9 ± 0.13 (median: 3) as opposed to 5.76 ± 0.25 (median: 5) for GT-P (Figure 2C).

In addition, after 7 days, Δ*dnaA* cells grown in low-phosphate displayed an average genome copy of 2.17 ± 0.10 (median: 2) per cell, compared to 2.94 ± 0.13 (median: 2) for GT-P, and the proportion of monoploid cells increased from 6 to 30%, respectively (Figures 2B,C). The average genome copy of GT-P cells grown in high-phosphate BG-11 was 4.51 ± 0.20 (median: 4), compared to 3.35 ± 0.15 (median: 3) for Δ*dnaA* cells, after 7 days of growth (Figure 2A).

A dramatic and significant difference was observed between GT-P and the Δ*dnaA* cells grown in low-phosphate BG-11 upon supplementation with phosphate at day 26 (Figures 3B,C). After 48 h, GT-P cells grown in low and no-phosphate BG-11 possessed an average genome copy number of 5.70 ± 0.25 (median: 5) and 6.13 ± 0.27 (median: 7), respectively, with over 50% of cells possessing >5 copies. Δ*dnaA* cells grown in low-phosphate media had an average genome copy of 2.92 ± 0.13, and a median value of 3 displayed by 67% of the population, and no cell contained more than eight genome copies, despite restoration of cell growth (Figure 3). Overall, DnaA seems to play a physiologically important role in re-establishing ploidy after phosphate starvation.

### Phosphate-Deprivation Natural Transformation Protocol for *Synechocystis* GT-P

Given that phosphate depletion leads to a significant reduction in median genome copy number and an increase in the proportion of monoploid cells (Figures 2B,C), we reasoned that growing cells under phosphate limiting conditions may decrease the time required to obtain fully segregated mutants.

To test this, we devised a simple transformation experiment in which cells were transformed with a plasmid that replaces the *psbD1/C* locus with a kanamycin-resistance cassette (Supplementary Figure 4). *psbD (sll0848)* encodes the D2 subunit of photosystem II and *psbC (sll0851)* encodes the CP43 apopolypeptide. The resulting fully segregated knockout mutant is incapable of growing photoautotrophically (Carpenter et al., 1990; Yu and Vermaas, 1990) but can be propagated photoheterotrophically in medium supplemented with 5 mM glucose. This means that partially segregated transformants will grow on both BG-11 and BG-11 + glucose plates whereas fully segregated transformants will only grow on BG-11 + glucose plates.

GT-P cells (∼1 × 10^9^) pre-grown in low-phosphate BG-11 were transformed and, after 24 h recovery on non-selective no-phosphate BG-11 agar plates, were incubated on selective no-phosphate BG-11 plates for 7 days. The number of kanamycin-resistant colonies obtained at this stage was approximately 100-fold less than obtained with the conventional transformation protocol using high-phosphate BG-11 medium but still gave hundreds of colonies (Supplementary Figure 5). Colonies were picked and streaked once again onto low-phosphate selective medium (containing 25 μg ml^–1^ kanamycin) and incubated for 7 days until single colonies appeared. Growth of the subsequent individual colonies was screened on BG-11 plates devoid of glucose.

**FIGURE 4:**
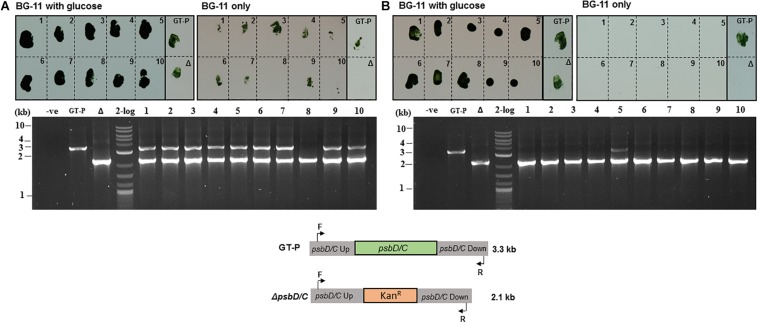
*Synechocystis* GT-P transformed using the **(A)** conventional and **(B)** phosphate-deprivation transformation protocol using a Δ*psbD1/C* gene-deletion construct. Ten kanamycin-resistant colonies obtained with each protocol (see “Materials and Methods”) were screened for growth on high-phosphate BG-11 plates with glucose or high-phosphate BG-11 only plates. PCR genotyping was used to test the segregation status of 10 colonies from **(A)** and **(B)**.

About 90% of the kanamycin-resistant colonies obtained using the conventional protocol maintained the ability to grow photoautotrophically suggesting they were still partially segregated, which was confirmed by colony-PCR genotyping, which showed that 9 out of 10 colonies grown on the BG-11 agar plate with 5 mM glucose possessed residual copies of the wild-type genome (Figure 4A). In contrast, using the phosphate deprivation protocol, none of the transformants was able to grow photoautotrophically after one round of selection and the *psbD1/C* operon could only be detected in one out of 10 transformants tested by PCR genotyping (Figure 4B).

**FIGURE 5:**
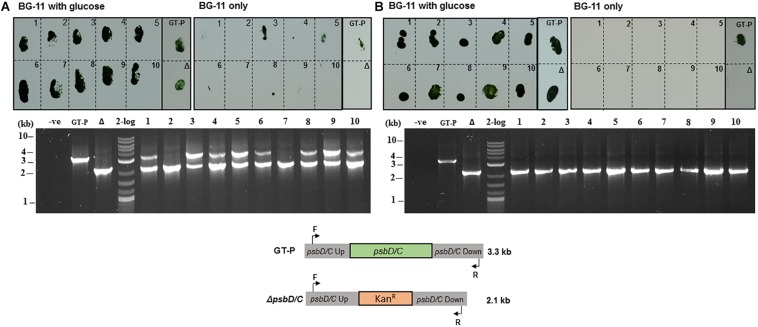
*Synechocystis*Δ*dnaA* transformed using **(A)** conventional and **(B)** phosphate-deprivation transformation protocol using a Δ*psbD1/C* gene-deletion construct. Ten kanamycin-resistant colonies obtained with each protocol (see materials and methods) were screened for growth on high-phosphate BG-11 plates with glucose or high-phosphate BG-11 only plates. PCR genotyping was used to test the segregation status of 10 colonies from **(A)** and **(B)**.

Approximately two out of 10 kanamycin-resistant colonies obtained after transforming the Δ*dnaA* mutant using the conventional protocol had segregated after one round of selection (Figure 5A), consistent with the reduced ploidy in this strain compared to GT-P cells (Figure 2). As expected, all colonies were fully segregated when using the phosphate deprivation natural transformation protocol (Figure 5B).

## Discussion

Using flow cytometry coupled with DNA staining, we report for the first time the population distribution of genome copies in the glucose-tolerant GT-P strain of *Synechocystis* sp. PCC 6803 and a Δ*dnaA* mutant grown under phosphate replete and starvation conditions. *Synechocystis* GT-P possesses a single circular chromosome of 3.57 Mbp and seven endogenous plasmids yielding a total genome size of 3.96 Mbp, assuming each endogenous plasmid is present at an equivalent ratio to the chromosome. Under our experimental conditions, GT-P cells grown in conventional BG-11 medium displayed average genome copy numbers of 6.05 ± 0.27, 4.51 ± 0.20, and 2.49 ± 0.11 (median values: 5, 3, and 2) during exponential, linear and stationary phases, respectively (Figure 2). Tichý et al. (2016) have previously used a similar approach to determine an average of 9 ± 2 copies per cell in the GT-P strain, but they did not specify the growth phase nor examine the population distribution.

qPCR and UV-spectroscopic methods used previously to determine genome copy number in *Synechocystis* have yielded a range of average values from 12 copies per cell in the wild-type PCC 6803 strain (Labarre et al., 1989) to average values of 142 ± 5 during exponential growth, 47 ± 7 during linear growth, and 43 ± 3 in stationary phase in a glucose-tolerant strain (Griese et al., 2011) and, in a later study, 15 ± 2, 7 ± 2, and 2 ± 1 in exponential, linear and stationary phases, respectively, for another glucose-tolerant strain (Kasuza sub-strain) (Zerulla et al., 2016). In the same study, the qPCR method was used to show that the average genome copy number of six different sub-strains of *Synechocystis* ranged from 30 to 2 genome copies per cell depending on sub-strain and growth phase (Zerulla et al., 2016).

The qPCR approach requires cell numbers to be determined prior to extraction of gDNA which is then quantified by qPCR methods, whereas the spectroscopic approach uses absorbance measurements of purified DNA at 260 nm to quantify DNA. Obviously, the accuracy of these approaches is dependent on reliable methods for both counting the number of cells within a population and for effective lysis of cells and downstream processing and quantification of DNA. A major disadvantage of these methods is that they do not provide information on the heterogeneity of genome copy number within the cell population.

We have demonstrated that a 10-fold reduction of phosphate in the BG-11 medium decreases the average genome copy number and increases the proportion of monoploid cells within a population. After 7 days in low-phosphate medium, *Synechocystis* GT-P possessed an average genome copy number of 2.94 ± 0.13 (median: 3) and 6% of the population (out of 50,000 analyzed) were monoploid compared to an average of 4.51 ± 0.2 (median: 4) and 0% monoploid cells when grown in high-phosphate BG-11 (Figure 2). A similar trend has previously been reported for the motile *Synechocystis* PCC 6803 strain which reduced its genome copy from 10 ± 2 (pre-culture) to 4 ± 2 (OD_740_ of 0.5) after 7 days of growth in the absence of phosphate (Zerulla et al., 2016).

Our work has also revealed a physiological role for the DnaA protein in regulating ploidy in *Synechocystis* GT-P. Our results indicate the Δ*dnaA* mutant can grow as well as GT-P in high-phosphate BG-11 but that growth is impaired in low and no-phosphate cultures (Figure 1B). Furthermore, when grown in low and no phosphate BG-11, the Δ*dnaA* mutant exhibited a greater proportion of monoploid cells (30% compared to 6% in GT-P) (Figures 1B,C). Compared to GT-P, the recovery of ploidy in Δ*dnaA* cells grown in low and no-phosphate BG-11 was also impaired after the addition of phosphate. Overall our results indicate a physiological role for DnaA in regulating DNA replication and controlling ploidy when grown under phosphate limitation.

We have exploited these observations to develop a new protocol for the efficient segregation of mutations in *Synechocystis* GT-P. Using the phosphate deprivation protocol, we have demonstrated that 90% of the analyzed transformants created in the GT-P background had fully segregated after two rounds of selection compared to 10% when using the conventional phosphate-replete protocol (Figures 4, 5). We hypothesize that the greater proportion of monoploid cells in GT-P cultures grown under phosphate deprivation and in Δ*dnaA* populations reduces the time needed to generate fully segregated mutants. More rapid segregation may also be promoted by the asymmetric distribution of genomes to daughter cells (Schneider et al., 2007; Jain et al., 2012). One disadvantage of the new method is the approximate 100-fold less efficient transformation efficiency (Supplementary Figure 5), possibly related to the differences in ploidy in the phosphate replete and depleted conditions. Nevertheless, if this reduction in transformation efficiency can be tolerated, then this protocol has the potential to reduce the time required to generate fully segregated mutants in *Synechocystis* GT-P, as well as possibly other cyanobacterial strains, which will be particularly useful for producing strains in the biotechnology area.

## Data Availability Statement

All datasets generated for this study are included in the article/Supplementary Material.

## Author Contributions

MP performed the experimental work. JH conducted all statistical analysis. MP and PN contributed to experimental design, data interpretation, and writing of the manuscript.

## Conflict of Interest

The authors declare that the research was conducted in the absence of any commercial or financial relationships that could be construed as a potential conflict of interest.
