# Supplementary material for: An Improved Natural Transformation Protocol for the Cyanobacterium Synechocystis sp. PCC 6803
Source: Front Plant Sci. 2020 Apr 15;11:372. doi: 10.3389/fpls.2020.00372 (PMC7174562; doi:10.3389/fpls.2020.00372)
Supplement: Supplementary file 1 [file Data_Sheet_1.PDF]

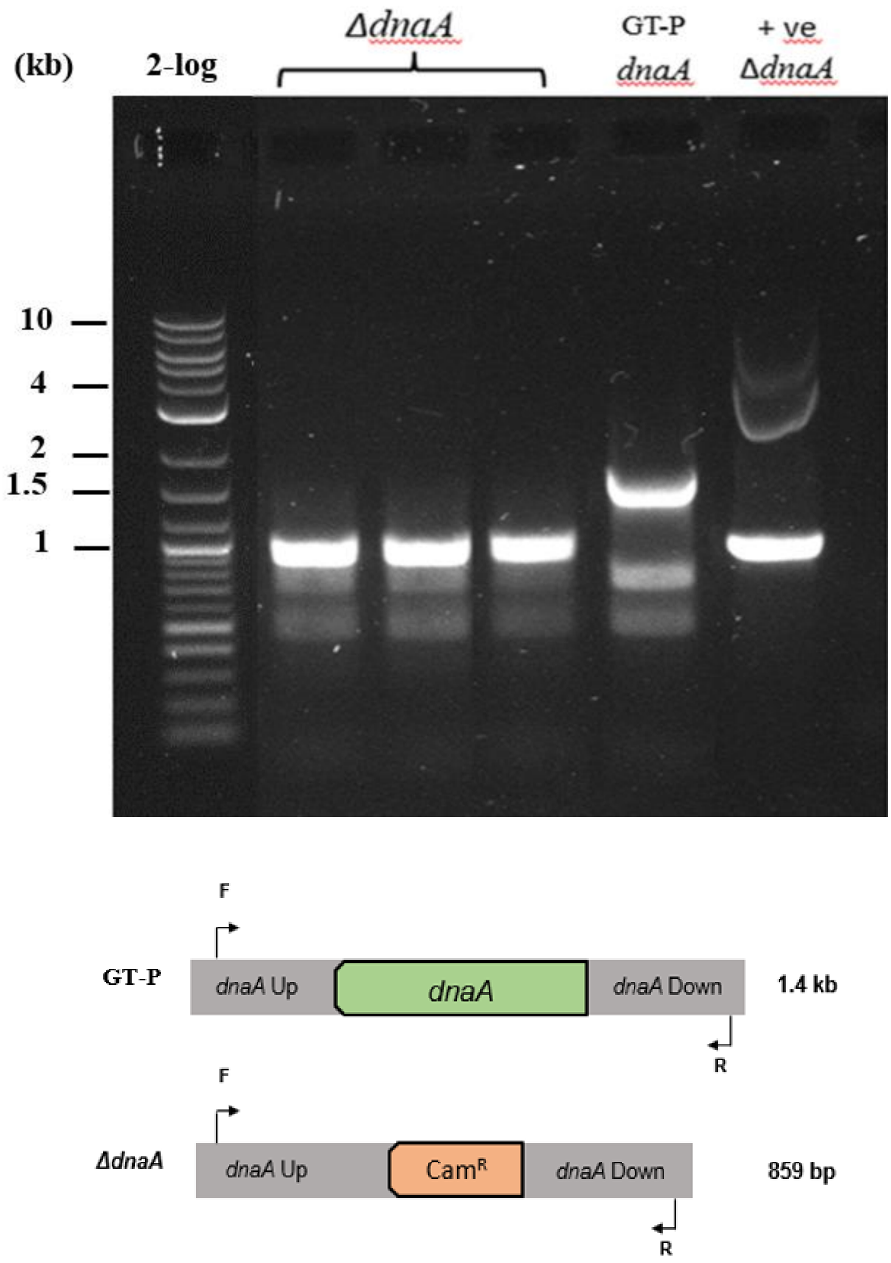

Supplementary Figure 1: PCR genotyping of three independent *ΔdnaA* mutants constructed in the *Synechocystis* GT-P strain. Forward (F) and reverse (R) primers were used for PCR analysis and fragments were separated by agarose gel electrophoresis. Predicted sizes of the PCR fragments are 1422 bp for the GT-P *dnaA* locus and 859 bp for the *ΔdnaA* locus.

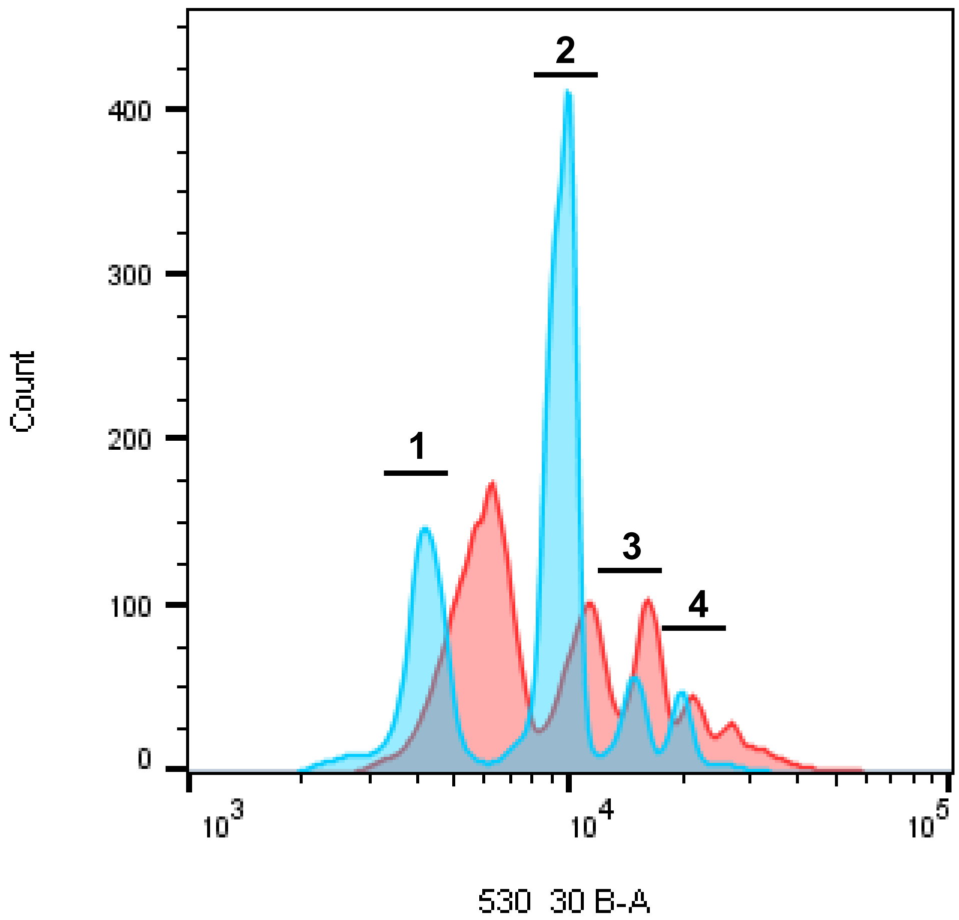

Supplementary Figure 2: Flow cytometry generated histogram representing the distribution of 9,000 *E. coli* cells grown in M9 minimal media before (red) and 60 minutes after (blue) the addition of chloramphenicol [20 µg µl^{-1}]. After chloramphenicol treatment, the average fluorescence intensity (FI) of cells assumed to be monoploid (1) is 4130 ± 494 arbitary units (A.U.). The average FI of cells assumed to contain 2 genome copies is 9,580 ± 350 (A.U.), 3 genome copies is 14,670 ± 596 (A.U.) and 4 genome copies is 19,810 ± 287 (A.U.). Cells possessing 5 or more genome copies do not appear after treatment with chloramphenicol. The fluorescence intensity of the cells assigned as monoploid (peak 1) after treatment with chloramphenicol corresponds to a genome size of 4.6 Mbp and is subsequently used as a reference to quantify the genome copy number of *Synechocystis* cells.

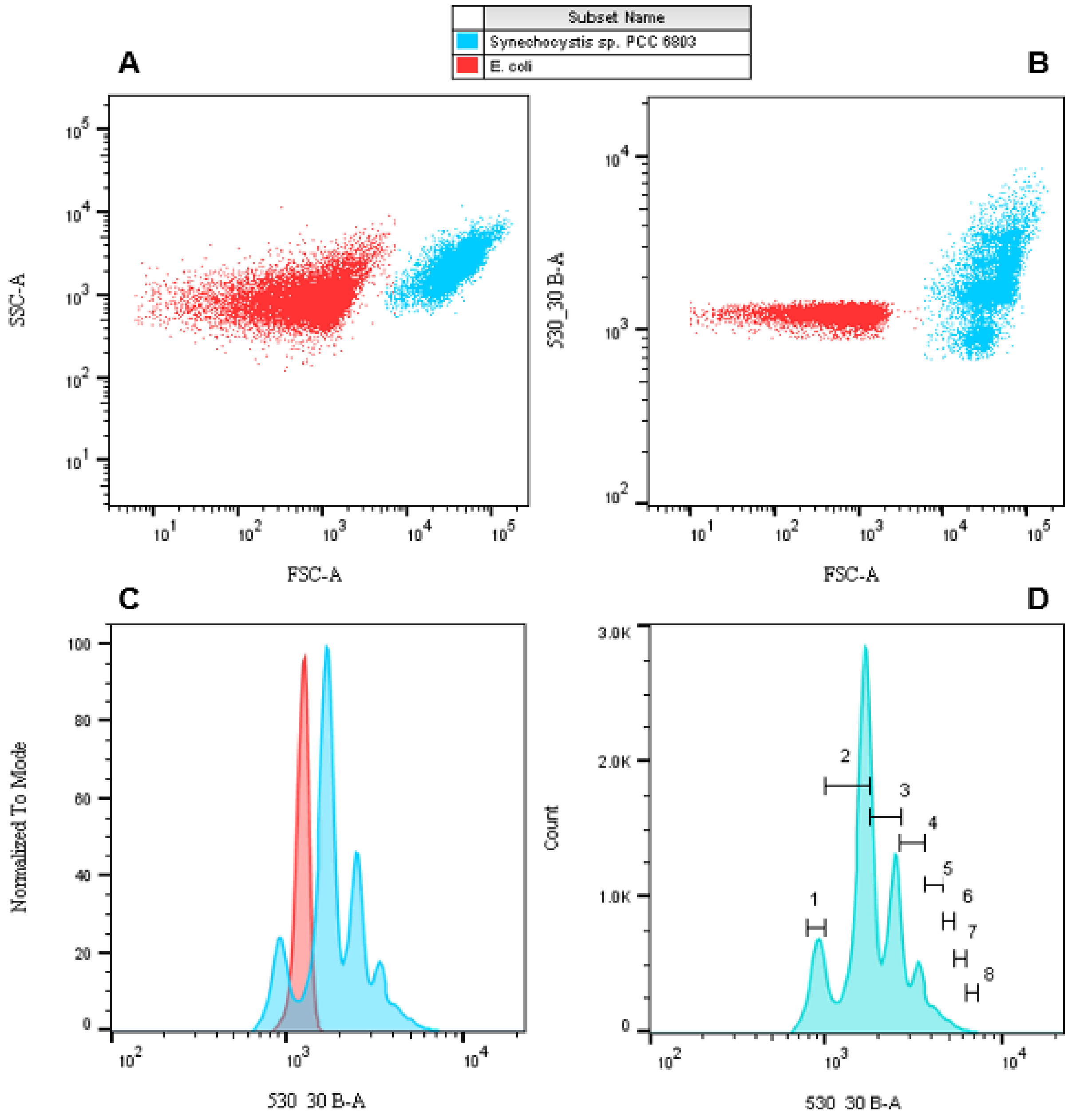

Supplementary Figure 3: Flow cytometric quantification of genome copy number in *Synechocystis* GT-P cells compared to *E. coli* reference. (A) A forward-scatter vs side-scatter dot-plot of *E. coli* (red) and *Synechocystis* (blue) cells. (B) A forward-scatter vs 530/30 nm (bandwidth) dot-plot of *E. coli* (red) and *Synechocystis* (blue) cells stained with the DNA fluorochrome, SYBR Green I. (C) A histogram overlaying the monoploid *E. coli* reference cells and *Synechocystis* cells reveals the distribution of genome copies within the *Synechocystis* population. (D) The fluorescence intensity (AU) per *Synechocystis* GT-P genome is deduced from the *E. coli* reference and gates are drawn over peaks in the emission spectra to distinguish genome copies per cell (Mori et al., 1996).

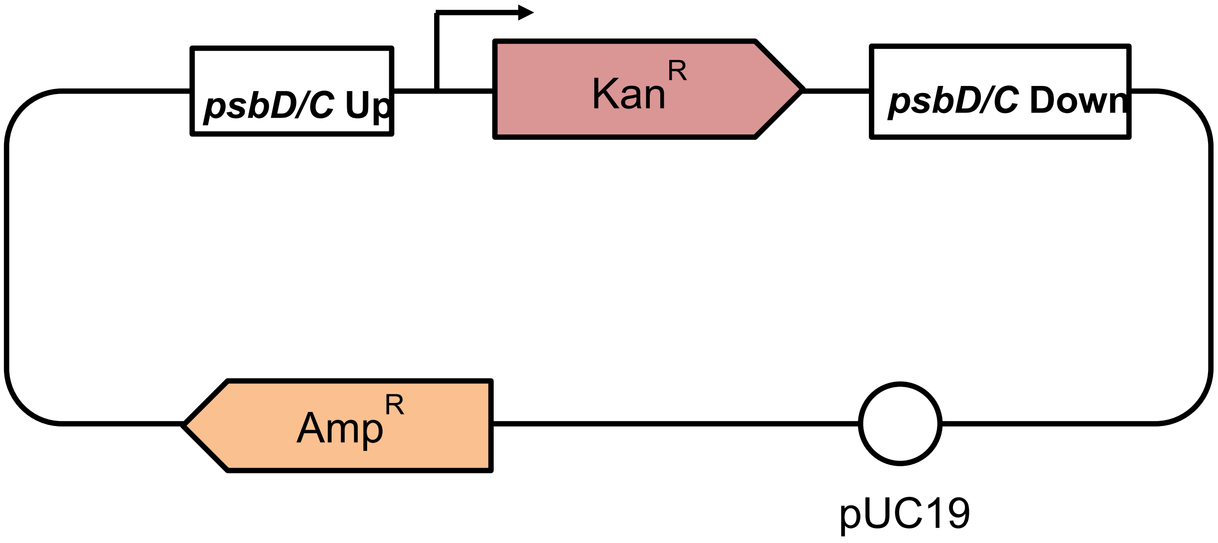

Supplementary Figure 4: Schematic of the *psbD/C* gene-deletion construct used in this study, kindly provided by Dr. Jiangfeng Yu, Imperial College London. Kanamycin (Kan^R) and ampicillin (Amp^R) resistance cassettes shown in coloured boxes, endogenous promoter indicated with black arrows, white boxes indicate regions of homology flanking *psbD1/C* flanking regions and white circle is pUC19 back-bone origin of replication.

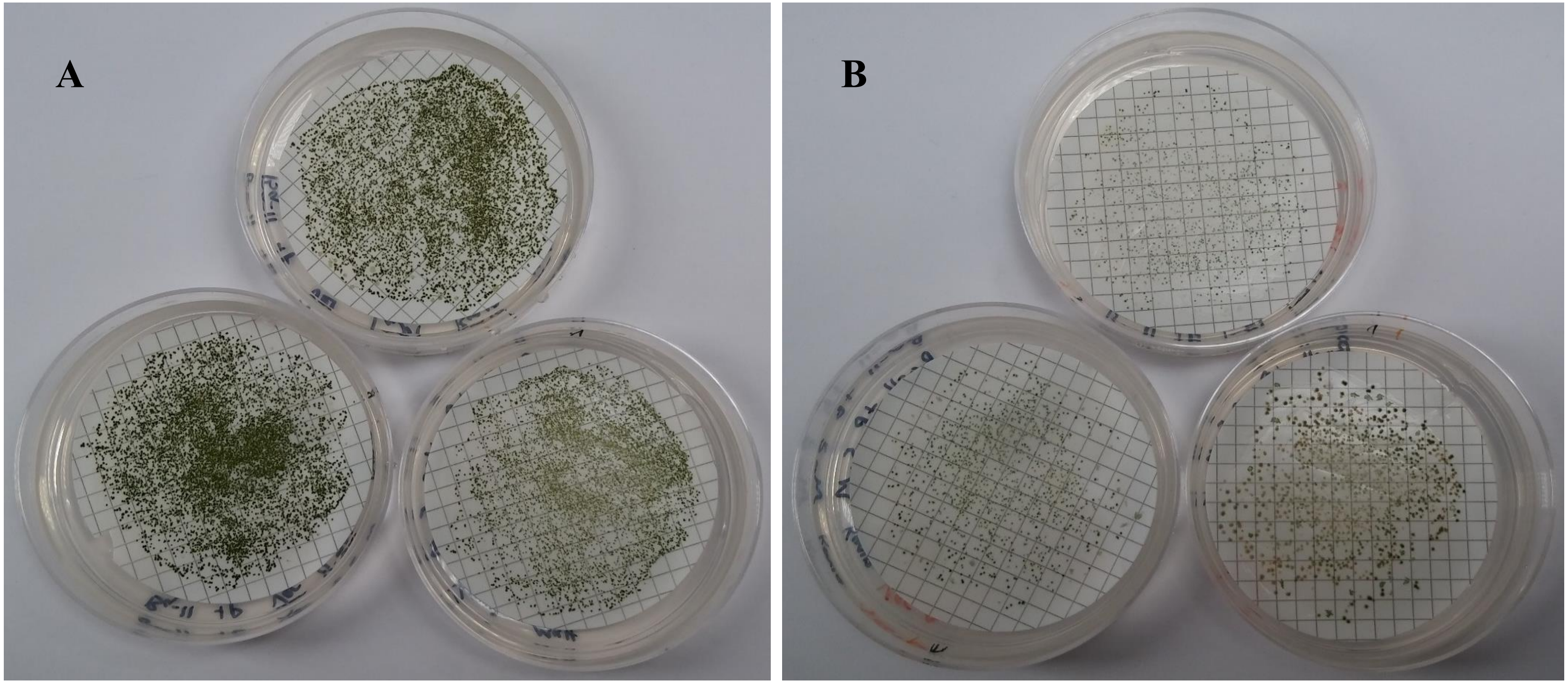

Supplementary Figure 5: Comparison between the transformation efficiency of *Synechocystis* GT-P using (A) the conventional phosphate-replete natural transformation protocol achieving 1 x 10^5 positive transformants per µg of plasmid DNA per OD_{740} of 1 and (B) the phosphate-deprivation natural transformation protocol. Cells were naturally transformed with 1 µg of *psbD/C* gene deletion construct (Supplementary Figure 4) with roughly 100-fold fewer positive transformants appearing after the initial round of selection.

Supplementary Table 1: Composition of 100X trace minerals required for M9 minimal media

**Trace minerals**	**Molecular weight**	**Concentration (g L^{-1})**
H_3BO_3	*61.83*	*0.1*
$MnCl_2*4H_2O$	*197.92*	*0.016*
$ZnCl_2$	*136.286*	*0.84*
$CuCl_2*2H_2O$	*134.45*	*0.13*
$CoCl_2*2H_2O$	*129.839*	*0.1*
$FeCl_3*6H2O$	*162.2*	*0.834*
EDTA	*292.24*	*5*
$_{dd}H_2O$	-	*To 1L*

* pH 7.5 – Filter sterilise

Supplementary Table 2: Glucose stock solution for M9 minimal media

**Constituent**	**Amount (g L^{-1})**
Glucose	*200*

* Autoclave for 15 minutes

Supplementary Table 3: $MgSO_4$ stock solution for M9 minimal media

**Constituent**	**Amount (g L^{-1})**
$MgSO_4*7H_2O$	*246.5*

* Autoclave for 15 minutes

Supplementary Table 4: $CaCl_2$ stock solution for M9 minimal media

**Constituent**	**Amount (g L^{-1})**
$CaCl_2$	*147*

***** Autoclave for 15 minutes

Supplementary Table 5: Composition of 1X M9 minimal media

**Constituent**	**Volume (ml)**
10xM9 salt solution	*100*
20% glucose	*20*
1M $MgSO_4$	*1*
1M $CaCl_2$	*0.3*
$_{dd}H_2O$	*To 1L*

* Filter sterilise if necessary

Supplementary Table 6: Composition of trace minerals required for BG-11 media

**Trace minerals**	**Molecular weight**	**Concentration (g L^{-1})**
H_3BO_3 Boric acid	*61.83*	*2.86*
*$MnCl_2$*4H_2O*	*197.92*	*1.81*
*$ZnSO_4$*7H_2O*	*287.54*	*0.222*
*Na_2MoO_4*2H_2O*	*241.96*	*0.390*
*$CuSO_4$*5H_2O*	*249.70*	*0.079*
*$Co(NO_3)_2$*6H_2O*	*291.05*	*0.0494*
$_{dd}H_2O$	-	*To 1L*

*Filter sterilise and store at 4ºC

Supplementary Table 7: Composition of 100X BG-FPC required for BG-11 media

**100X BG-FPC**	**Molecular weight**	**Concentration (g L^{-1})**
$NaNO_3$	*84.99*	*149.58*
*$MgSO_4$*7H_2O*	*246.47*	*7.5*
$CaCl_2$	*110.98*	*2.72*
Citric acid	*192.13*	*0.60*
Na_2EDTA pH = 8.0	-	*1.12 ml*
Trace minerals	-	*100 ml*
$_{dd}H_2O$	-	*To 1 L*

*Store at -25ºC

Supplementary Table 8: Composition of Ferric ammonium citrate stock solution in H_2O

Stock	Concentration (mM)
1000X Ferric ammonium citrate	*42*

* Filter sterilised, stored in dark at 4°C

Supplementary Table 9: Composition of Na_2CO_3 stock solution in H_2O

Stock	Concentration (mM)
1000X Na_2CO_3	*189*

* Filter sterilised, stored at 4°C

Supplementary Table 10: Composition of K_2HPO_4 stock solution in H_2O

Stock	Concentration (mM)
1000X K_2HPO_4	*175*

* Filter sterilised, stored at 4°C

Supplementary Table 11: Composition of KCl stock solution in H_2O

Stock	Concentration (mM)
1000X KCl	*175*

* Filter sterilised, stored at 4°C

Supplementary Table 12: Composition of 1 litre of high-phosphate BG-11 medium

Stock	Volume (ml)	Concentration (mM)
1 M TES-KOH, pH=8.2	*5 ml*	*5 mM*
100X BG-FPC	*10 ml*	-
1000X Ferric ammonium citrate	*1 ml*	*0.042 mM*
1000X Na_2CO_3	*1 ml*	*0.189 mM*
1000X K_2HPO_4	*1 ml*	*0.175 mM*
$_{dd}H_2O$	*To 1 L*	-

* Autoclave for 25 minutes

Supplementary Table 13: Composition of 1 litre of low-phosphate BG-11 media.

Stock	Volume (ml)	Concentration (mM)
1 M TES-KOH, pH=8.2	*5 ml*	*5 mM*
100X BG-FPC	*10 ml*	-
1000X Ferric ammonium citrate	*1 ml*	*0.042 mM*
1000X Na_2CO_3	*1 ml*	*0.189 mM*
1000X KCl	*0.9 ml*	*0.157 mM*
1000X K_2HPO_4	*0.1 ml*	*0.0175 mM*
$_{dd}H_2O$	*To 1 L*	-

* Autoclave for 25 minutes

No phosphate BG-11

Supplementary Table 14: Composition of 1 litre of no-phosphate BG-11 media.

**Stock**	**Volume (ml)**	**Concentration (mM)**
1 M TES-KOH, pH=8.2	*5 ml*	*5 mM*
100X BG-FPC	*10 ml*	-
1000X Ferric ammonium citrate	*1 ml*	*0.042 mM*
1000X Na_2CO_3	*1 ml*	*0.189 mM*
1000X KCl	*1 ml*	*0.175 mM*
$_{dd}H_2O$	*To 1 L*	-

* Autoclave for 25 minutes

Supplementary Table 15: Composition of solid high-phosphate BG-11 medium.

**Stock**	**Volume/Weight**
Difco Bacto Agar	*4 g*
High-phosphate liquid BG-11 (1x)	*Fill to 333 ml*

* Autoclave for 25 minutesLow phosphate solid BG-11

Supplementary Table 16: Composition of solid low-phosphate BG-11 medium.

**Stock**	**Volume/Weight**
Difco Bacto Agar	*4 g*
Low phosphate liquid BG-11 (1x)	*Fill to 333 ml*

* Autoclave for 25 minutes

Supplementary Table 17: Composition of solid no-phosphate BG-11 medium.

Stock	Volume/Weight
Difco Bacto Agar	*4 g*
Liquid no-phosphate BG-11 (1x)	*Fill to 333 ml*

* Autoclave for 25 minutes

Supplementary Table 18: Stock and working concentrations of all antibiotics used in this study along with the solvents they were dissolved in.

**Antibiotic**	**Solvent**	**Stock [ ] (μg ml^{-1})**	**Working concentration *Synechocystis* GT-P (μg ml^{-1})**	**Working concentration *E. coli* (μg ml^{-1})**
Kanamycin	$_dH_2O$	*100*	*5-100*	*50-100*
Chloramphenicol	*50-100% ethanol*	*50*	*25-100*	*25-50*

Supplementary Table 19: Results of the linear mixed model detailing the parameter estimates, standard errors, degrees of freedom and associated *t*-values. The R^2 of the model was 0.52. The random intercept – *replicate* – was found to explain <1% of the variation in log fluorescence (A.U.).

	**Estimate**	**Std. Error**	**Degrees of Freedom**	***t*-value**
(Intercept)	9.81	0.02	4.07	627.58
GT-P	0.13	0.02	4.07	5.69
Day 7	-0.47	0.00	3333611.35	-336.17
Day 21	-0.87	0.00	3333606.50	-607.13
Day 26	-0.90	0.00	3333611.05	-550.92
Day 28	-0.63	0.00	3333611.96	-372.24
Low-Phosphate	0.05	0.00	3333610.66	29.77
No-Phosphate	-0.61	0.00	3333611.99	-359.57
GT-P:Day 7	0.17	0.00	3333612.00	73.33
GT-P:Day 21	-0.02	0.00	3333611.66	-8.09
GT-P:Day 26	0.10	0.00	3333611.17	38.11
GT-P:Day 28	-0.13	0.00	3333612.00	-52.22
GT-P:Low-Phosphate	0.03	0.00	3333596.58	10.35
GT-P:No-Phosphate	0.56	0.00	3333610.99	196.09
Day 7: Low-Phosphate	-0.48	0.00	3333610.31	-256.28
Day 21: Low-Phosphate	-0.27	0.00	3333606.69	-138.13
Day 26: Low-Phosphate	-0.21	0.00	3333608.67	-94.83
Day 28: Low-Phosphate	-0.02	0.00	3333611.91	-6.37
Day 7:No-Phosphate	0.08	0.00	3333611.62	43.44
Day 21:No-Phosphate	0.57	0.00	3333612.00	279.63
Day 26:No-Phosphate	0.61	0.00	3333611.55	266.06
Day 28:No-Phosphate	0.55	0.00	3333610.84	226.40
GT-P:Day 7:Low-Phosphate	-0.02	0.00	3333609.15	-6.20
GT-P:Day 21:Low-Phosphate	0.33	0.00	3333604.03	98.97
GT-P:Day 26:Low-Phosphate	0.19	0.00	3333611.34	53.33
GT-P:Day 28:Low-Phosphate	0.65	0.00	3333607.30	171.03
GT-P:Day 7:No-Phosphate	-0.47	0.00	3333611.98	-134.63
GT-P:Day 21:No-Phosphate	-0.38	0.00	3333611.75	-100.55
GT-P:Day 26:No-Phosphate	-0.48	0.00	3333612.00	-127.64
GT-P:Day 28:No-Phosphate	0.28	0.00	3333611.86	73.22

Supplementary Table 20: The least square means and 95% confidence intervals estimated by the linear mixed model (Table 19) and their approximate number of genome copies.

	**Log FI Estimate±95% CI**	**Genome Copy±95% CI**	**Degrees of Freedom**	***t*-value**
ΔdnaA	9.1±0.04	2.63±0.12	4.03	583.97
GT-P	9.45±0.04	3.74±0.17	4.02	606.40
Day 2	9.78±0.03	5.20±0.16	4.04	886.54
Day 7	9.19±0.03	2.87±0.09	4.03	833.15
Day 21	8.99±0.03	2.36±0.07	4.03	815.21
Day 26	9.01±0.03	2.41±0.07	4.03	817.04
Day 28	9.41±0.03	3.60±0.11	4.04	853.19
High-Phosphate	9.31±0.03	3.23±0.10	4.03	844.18
Low-Phosphate	9.29±0.03	3.17±0.10	4.03	842.32
No-Phosphate	9.23±0.03	3.01±0.09	4.03	837.64
ΔdnaA:Day 2	9.62±0.04	4.43±0.20	4.04	616.83
GT-P: Day 2	9.94±0.04	6.10±0.27	4.04	636.93
ΔdnaA:Day 7	9.02±0.04	2.43±0.11	4.03	578.79
GT-P: Day 7	9.35±0.04	3.38±0.15	4.03	599.45
ΔdnaA:Day 21	8.85±0.04	2.04±0.09	4.03	567.56
GT-P: Day 21	9.13±0.04	2.72±0.12	4.03	585.31
ΔdnaA:Day 26	8.85±0.04	2.05±0.09	4.04	567.56
GT-P: Day 26	9.17±0.04	2.83±0.12	4.03	587.91
ΔdnaA:Day 28	9.16±0.04	2.81±0.12	4.05	587.31
GT-P: Day 28	9.66±0.04	4.61±0.20	4.03	619.30
ΔdnaA:High-Phosphate	9.23±0.04	3.00±0.13	4.03	592.22
GT-P:High-Phosphate	9.38±0.04	3.49±0.15	4.02	601.62
ΔdnaA:Low-Phosphate	9.08±0.04	2.59±0.11	4.03	582.70
GT-P:Low-Phosphate	9.49±0.04	3.88±0.17	4.02	608.51
ΔdnaA:No-Phosphate	8.98±0.04	2.35±0.10	4.03	576.45
GT-P:No-Phosphate	9.48±0.04	3.87±0.17	4.03	608.14
Day 2:High-Phosphate	9.87±0.03	5.68±0.18	4.07	892.72
Day 7:High-Phosphate	9.49±0.03	3.88±0.12	4.05	859.34
Day 21:High-Phosphate	8.99±0.03	2.36±0.07	4.06	814.01
Day 26:High-Phosphate	9.01±0.03	2.42±0.07	4.07	815.60
Day 28:High-Phosphate	9.17±0.03	2.82±0.09	4.07	829.46
Day 2:Low-Phosphate	9.93±0.03	6.04±0.19	4.07	898.39
Day 7:Low-Phosphate	9.06±0.03	2.53±0.08	4.05	820.59
Day 21:Low-Phosphate	8.94±0.03	2.25±0.07	4.05	809.86
Day 26:Low-Phosphate	8.96±0.03	2.29±0.07	4.06	811.18
Day 28:Low-Phosphate	9.54±0.03	4.08±0.13	4.10	861.45
Day 2:No-Phosphate	9.54±0.03	4.09±0.13	4.10	861.76
Day 7:No-Phosphate	9.01±0.03	2.40±0.07	4.05	816.04
Day 21:No-Phosphate	9.04±0.03	2.47±0.08	4.08	817.29
Day 26:No-Phosphate	9.06±0.03	2.52±0.08	4.06	819.72
Day 28:No-Phosphate	9.53±0.03	4.05±0.13	4.07	862.15
ΔdnaA:Day 2:High-Phosphate	9.81±0.04	5.34±0.23	4.07	627.58
GT-P: Day 2:High-Phosphate	9.93±0.04	6.05±0.27	4.08	634.92

ΔdnaA:Day 7:High-Phosphate	9.34±0.04	3.35±0.15	4.04	598.78
GT-P: Day 7:High-Phosphate	9.64±0.04	4.51±0.20	4.06	616.49
ΔdnaA:Day 21:High-Phosphate	8.94±0.04	2.23±0.10	4.05	572.73
GT-P: Day 21:High-Phosphate	9.04±0.04	2.49±0.11	4.06	578.45
ΔdnaA:Day 26:High-Phosphate	8.9±0.04	2.16±0.10	4.07	569.90
GT-P: Day 26:High-Phosphate	9.13±0.04	2.70±0.12	4.07	583.53
ΔdnaA:Day 28:High-Phosphate	9.17±0.04	2.83±0.12	4.08	586.89
GT-P: Day 28:High-Phosphate	9.16±0.04	2.81±0.12	4.07	586.15
ΔdnaA:Day 2:Low-Phosphate	9.85±0.04	5.59±0.25	4.06	630.97
GT-P: Day 2:Low-Phosphate	10.01±0.04	6.51±0.29	4.08	639.55
ΔdnaA:Day 7:Low-Phosphate	8.91±0.04	2.17±0.10	4.04	571.11
GT-P: Day 7:Low-Phosphate	9.21±0.04	2.94±0.13	4.06	589.37
ΔdnaA:Day 21:Low-Phosphate	8.71±0.04	1.79±0.08	4.05	558.41
GT-P: Day 21:Low-Phosphate	9.17±0.04	2.83±0.12	4.06	586.88
ΔdnaA:Day 26:Low-Phosphate	8.74±0.04	1.84±0.08	4.06	559.98
GT-P: Day 26:Low-Phosphate	9.18±0.04	2.84±0.13	4.06	587.18
ΔdnaA:Day 28:Low-Phosphate	9.2±0.04	2.92±0.13	4.13	587.01
GT-P: Day 28:Low-Phosphate	9.87±0.04	5.70±0.25	4.07	631.33
ΔdnaA:Day 2:No-Phosphate	9.2±0.04	2.90±0.13	4.07	588.60
GT-P: Day 2:No-Phosphate	9.88±0.04	5.76±0.25	4.12	630.03
ΔdnaA:Day 7:No-Phosphate	8.82±0.04	1.98±0.09	4.04	565.34
GT-P: Day 7:No-Phosphate	9.2±0.04	2.91±0.13	4.06	588.68
ΔdnaA:Day 21:No-Phosphate	8.89±0.04	2.14±0.09	4.05	570.07
GT-P: Day 21:No-Phosphate	9.18±0.04	2.86±0.13	4.11	585.72
ΔdnaA:Day 26:No-Phosphate	8.9±0.04	2.16±0.10	4.06	570.19
GT-P: Day 26:No-Phosphate	9.21±0.04	2.93±0.13	4.07	589.05
ΔdnaA:Day 28:No-Phosphate	9.11±0.04	2.67±0.12	4.08	583.15
GT-P: Day 28:No-Phosphate	9.94±0.04	6.13±0.27	4.07	636.10

Supplementary Table 21: Results of the pairwise comparisons of least square means (Table-20) with their estimate difference and 95% confidence intervals. A *t*-value less than -16.25 and greater than 16.25 was interpreted as significant to correct for multiple testing.

	**Estimated Difference [95% CI]**	**Degrees of Freedom**	***t*-value**
Day 2			
High-Phosphate vs Low-Phosphate	-0.060 [-0.063, -0.058]	3333596.58	-47.37
High-Phosphate vs No-Phosphate	0.329 [0.326, 0.332]	3333610.99	230.93
Low-Phosphate vs No-Phosphate	0.389 [0.387, 0.392]	3333609.93	277.72
Day 7			
High-Phosphate vs Low-Phosphate	0.430 [0.428, 0.432]	3333608.06	424.38
High-Phosphate vs No-Phosphate	0.480 [0.478, 0.482]	3333608.10	471.17
Low-Phosphate vs No-Phosphate	0.050 [0.048, 0.052]	3333608.13	50.24
High-Phosphate			
Day 26 vs 2 Days After Phosphate Addition	-0.154 [-0.156, -0.152]	3333608.6	-122.80
Low-Phosphate			
Day 26 vs 2 Days After Phosphate Addition	-0.577 [-0.580, -0.575]	3333610.85	-428.87
No-Phosphate			
Day 26 vs 2 Days After Phosphate Addition	-0.473 [-0.476, -0.471]	3333609.66	-389.20
ΔdnaA			
Day 26 vs 2 Days After Phosphate Addition	-0.313 [-0.316, -0.311]	3333609.78	-306.30
GT-P			
Day 26 vs 2 Days After Phosphate Addition	-0.490 [-0.492, -0.488]	3333609.30	-463.98
High-Phosphate Day 2			
ΔdnaA vs GT-P	-0.126 [-0.187, -0.065]	4.07	-5.69
Low-Phosphate Day 2			
ΔdnaA vs GT-P	-0.152 [-0.091, 0.004]	4.07	-6.88
No-Phosphate Day 2			
ΔdnaA vs GT-P	-0.684 [-0.745, -0.623]	4.10	-30.91
High-Phosphate Day 7			
ΔdnaA vs GT-P	-0.298 [-0.237, 0.000]	4.05	-13.49
Low-Phosphate Day 7			
ΔdnaA vs GT-P	-0.304 [-0.365, -0.243]	4.05	-13.77
No-Phosphate Day 7			
ΔdnaA vs GT-P	-0.384 [-0.446, -0.324]	4.05	-17.44
High-Phosphate 2 Days After Phosphate Addition			
ΔdnaA vs GT-P	0.008 [-0.053. 0.069]	4.07	0.36
Low-Phosphate 2 Days After Phosphate Addition			
ΔdnaA vs GT-P	-0.669 [-0.730, -0.608]	4.10	-30.22
No-Phosphate 2 Days After Phosphate Addition			
ΔdnaA vs GT-P	-0.830 [-0.891, -0.769]	4.07	-37.53

Supplementary Table 22: Fluorescence intensity readings and their corresponding genome copy numbers in *Synechocystis* GT-P deduced from an average fluorescence intensity of 4130 ± 494 from *E. coli* reference cells assigned monoploidy and the lowest reading of 3565 ± 85 for *Synechocystis* GT-P cells assigned monoploidy. Any cells possessing less than half a genome were rounded down, conversely, any cells possessing more than half a genome were rounded up.

**FI (A.U.)**	**Actual Genome copy number**	**Assigned genome copy number**
3400-5100	0.95-1.5	1
5100-8500	1.5-2.5	2
8500-11900	2.503.5	3
11900-15300	3.5-4.5	4
15300-18700	4.5-5.5	5
18700-22100	5.5-6.5	6
22100-25500	6.5-7.5	7
25500-28900	7.5-8.5	8
28900-32300	8.5-9.5	9
32300-35700	9.5-10.5	10
35700-39100	10.5-11.5	11
39100-42500	11.5-12.5	12
42500-45900	12.5-13.5	13
45900-47600	13.5-14.5	14
47600-	14.5-	15+
